# Differentiation of adult human retinal pigment epithelial cells into dopaminergic-like cells in vitro and in the recipient monkey brain

**DOI:** 10.1186/s10020-019-0076-3

**Published:** 2019-03-29

**Authors:** Sha Li, Han Zhang, Aifang Wang, Yan Liu, Houqi Liu, Feng Yue, Xianmixinuer Abulaiti, Caiqiao Zhang, Lingsong Li

**Affiliations:** 1 College of Animal Sciences, Zhejiang University, Hangzhou, 310029 China; 2 Shanghai Advanced Research Institute, Chinese Academy of Sciences, Shanghai, 201210 China; 3 University of Chinese Academy of Sciences, Beijing, 100049 China; 4 School of Life Science and Technology, ShanghaiTech University, Shanghai, 201210 China; 5 Institute of Biochemistry and Cell Biology, Chinese Academy of Sciences, Shanghai, 201210 China; 6 Research Center of Developmental Biology, Second Military Medical University, Shanghai, 200433 China; 7 Department of Neurobiology, Xuanwu Hospital of Capital Medical University, Beijing, 100053 China; 8 Henan Key laboratory of Stem Cell Differentiation and Modification, Henan Provincial People’s Hospital, Henan University and Zhengzhou University, Zhengzhou, China

**Keywords:** Cell-replacement therapy, Parkinson’s disease, Retinal pigment epithelium, Chemically induced reprogramming

## Abstract

**Background:**

Cell therapy is proposed to be a potential treatment for Parkinson’s disease (PD). Although fetal retinal pigment epithelial (RPE) cells have been tested in trials for treating PD patients, controversy has been raised over the issue of whether such cells can be reprogrammed into dopamine-producing cells for therapeutic efficacy. Here, we aim to investigate whether adult human RPE cells can be reprogrammed into dopamine-producing cells both in vitro and in the recipient monkey brain.

**Methods:**

The RPE layer was isolated from frozen posterior eyeball tissue after penetrating keratoplasty surgery. The tumorigenicity of RPE cells was examined by G-banding and a tumor formation assay in nude mice. Immunogenicity was measured using a one-way mixed lymphocyte reaction (MLR) assay. Dopamine-production in chemically reprogrammed RPE cells was measured by HPLC. Finally, RPE cells were grafted into the brains of monkeys with MPTP-induced PD in order to investigate the potential of such cells treating PD patients in the future.

**Results:**

RPE cell lines have been successively established from adult human eye tissues. Such cells can be chemically reprogrammed into dopamine-producing cells in vitro. Moreover, after being grafted into the brain caudate putamen of monkeys with MPTP-induced PD, RPE cells became tyrosine hydroxylase-positive cells, and recipient PD monkeys showed significant improvement of clinical conditions.

**Conclusions:**

This preclinical study using a primate model indicates that human adult RPE cells could be a potential cell source for the treatment of PD in the future.

**Electronic supplementary material:**

The online version of this article (10.1186/s10020-019-0076-3) contains supplementary material, which is available to authorized users.

## Background

Parkinson’s disease (PD) is one of the most common neurodegenerative diseases among the elderly population. Patients with PD typically suffer from resting tremors, rigidity, hypokinesia, disturbances in gait, balance and autonomic functions, depression, and dementia. From an etiological perspective, PD is caused by the loss of dopamine (DA)-producing neurons in the substantia nigra pars compacta (Hardy, 2010). This substantia nigra–striatum neuronal projection is responsible for the motor symptoms during disease. Starting from an alteration in the midbrain DA neurons, the disease will eventually affect forebrain neurons, such as the cortical neurons. Several susceptibility factors have been identified in the last decade (Chung et al., 2002; Lesage and Brice, 2009). These studies suggest that protein misfolding, an abnormal increase in oxidative stress, mitochondrial dysfunction, and impairments in the ubiquitin–proteasome and autophagy-lysosomal systems can all contribute to the development of PD.

Patients with PD are currently treated with a variety of pharmacological drugs, including levodopa, dopamine agonists, and monoamine oxidase B inhibitors. Among them, levodopa is the most effective drug, but unwanted side effects are often observed, including fluctuations in the symptoms of PD, and dyskinesias (Chapuis et al., 2005; Isacson, 2004). Bilateral subthalamic nucleus or globus pallidus internus (GPi) deep brain stimulations (DBS) can improve the condition of patients, reduce their daily dose of levodopa, and ameliorate levodopa-related motor complications and dyskinesias. Nonetheless, DBS was reported to cause severe depression (Kriks et al., 2011). In spite of improvements in symptom management, all of these therapies cannot stop disease progression (Krack et al., 2003).

In the hopes of stopping disease progression by replacing diseased neurons, cell therapy has been tested for treating animals with PD or patients with PD in clinical trials. In this regard, fetal-derived neural stem cells (NSCs) were the first cell type to be investigated, followed by mesenchymal stem cells, dopaminergic neurons differentiated from embryonic stem (ES) cells, and induced pluripotent stem (iPS) cells, respectively (Ambasudhan et al., 2014; Ben-Hur et al., 2004; Li et al., 2001; McGuckin et al., 2004; Park et al., 2008; Roy et al., 2006; Weiss et al., 2006; Yang et al., 2008). However, safety issues, ethical concerns, and capacities for dopamine-production in over-grafted cells have limited clinical application for patients with PD. To look for alternative cell sources for PD treatment, an open label pilot study using human fetal RPE cells was reported to be able to improve patients’ conditions, as observed up to 12 months after transplantation (Stover et al., 2005). However, a randomized double-blinded study using the same RPE cells did not confirm the same therapeutic effect (Katsnelson, 2011). In addition, there are ethical concerns that remain regarding the utilization of fetal tissues for clinic use.

In this report, we have established RPE cell lines from adult human eye tissues. Moreover, such cells can be chemically reprogrammed into dopamine-producing neuron-like cells in vitro. After being grafted into the brain caudate putamen of monkeys with chemically induced PD, RPE-grafted PD monkeys showed significant improvement of clinical conditions, due to the fact that some transplanted RPE cells became positive for tyrosine hydroxylase. We therefore propose that adult human RPE cells can be an alternative cell source for the treatment of PD in the future.

## Methods

### Isolation and culture of human RPE cells

Human eyes from a 57-year-old donor were obtained from Beijing Tongren Eye Bank (a member of the International Association of Eye Banks). The posterior eyeball tissues that remained after penetrating keratoplasty surgery were used to isolate the layer of RPE cells. The tissues were sliced at the ora serrata, and the anterior parts and vitreous were discarded, which exposed the RPE layer. The RPE layers were dissected into small debris and plated into a 6-well plate with minimal contamination by rod outer segments, blood, or other cell types. RPE cells were cultured in medium containing MEM-a modified medium (Sigma-Aldrich, St. Louis, MO, USA), 2 mM L-glutamine, penicillin/streptomycin (1:100), 1% Na-Pyruvate, and 10% FBS (fetal bovine serum). Cells were incubated in a 37 °C, 5% CO_2_ humidified incubator, and the medium was replaced every 3 days.

When the cultures reach approximately 80% confluency remove the medium from the well and wash with PBS. (Do not allow the cells to exceed 90% confluency as this may lead to detachment of cells and increased levels of cell death). Add 200 μl accutase and incubate at 37 °C for 2–3 min (checking to see if the cells are rounded and detached). Remove the cells to a 15 ml tube and wash the well once with PBS and transfer to the same tube. Dilute cells to 5 ml with PBS and centrifuge 300 x g for 5 min. And for passage, suspend the cells in 200 μl medium, and plated onto 6-well plates at a density of 2× 104 cells per well.

### Induction of dopamine-producing cells from RPE cells

RPE cells were plated onto 6-well plates at a density of 2 × 10^4^ cells per well in neural differentiation medium containing neurobasal medium with B27 supplement (Thermo Fisher Scientific, Waltham, MA, USA), 2 mM l-glutamine (Thermo Fisher Scientific, Waltham, MA, USA), 1 mM sodium pyruvate (Sigma-Aldrich, St. Louis, MO, USA), 0.1 mM 2-mercaptoethanol, 100 nM LDN193189 (Stemgent, Cambridge, MA, USA), 10 μM CHIR99021 (Stemgent, Cambridge, MA, USA), and 10 μM SB431542 (Stemgent, Cambridge, MA, USA).During the initial first passage, overnight treatment with a ROCK inhibitor (Y-27632, 4 μM, Stemgent, Cambridge, MA, USA) was used to enhance cell survival, but was not required in following passages. After that, cells were passaged after 3 days. The cells were cultured in neuronal induction medium until day 7.

### Immunofluorescence microscopy

Cells on coverslips were fixed in cold 4% PFA for 15 min. After three washes in phosphate buffered saline, cells were blocked with phosphate buffered saline containing 5% bovine serum albumin for 30 min and then incubated with the appropriate primary antibody for 12 h at 4 °C. For histological studies, animals were perfused with 100 mM PBS and then 4% paraformaldehyde in PBS following an intravenous infusion of Potassium Chloride (KCl) (75–150 mg/kg) after general anesthesia. Brains were post-fixed for 1 day with 4% paraformaldehyde and then transferred to 10, 20 and 30% sucrose solution at 4 °C. The brains were sectioned at 40-μm thickness using a microtome. Histochemical analyses were carried out after permeabilization and blocking with phosphate buffered saline containing 5% bovine serum albumin for 30 min. Primary antibodies used in this study include the following: rabbit anti-Ki67 (Thermo Fisher Scientific, Waltham, MA, USA; MA5–1452), mouse anti-RPE65 (NOVUSBIO, Centennial, CO, USA; NB100-355ss), rabbit anti-Pax6 (Abcam, Cambridge, MA, USA; ab179760), mouse anti-Bestrophin1 (NOVUSBIO, Centennial, Colorado, USA; NB300-164ss), rabbit anti-Nurr1 (Santa Cruz, Santa Cruz, CA, USA; sc-365,509), mouse anti-CK18 (Abcam, Cambridge, MA, USA; ab668), mouse anti-FOXA2 (ABGENT, San Diego, CA, USA; AP12031b), rabbit anti-VMAT2 (Proteintech, Chicago, IL, USA;20,873–1-AP), rabbit anti-DAT (Proteintech, Chicago, IL, USA; 22,524–1-AP), anti- rabbit GRIK2 (Proteintech, Chicago, IL, USA; 13,597–1-AP), rabbit anti-Calbindin (Proteintech, Chicago, IL, USA; 10,166–1-AP), and mouse anti-TH (Santa Cruz, Santa Cruz, CA, USA; sc-20,088). Primary antibodies were used at a 1:200 dilution in solution A (Toyobo, Osaka, Japan; NKB-501). After three washes, cells were incubated with goat secondary antibodies conjugated to Alexa Flour 488 or Alexa Flour 594 in blocking buffer for 1 h at 37 °C and then with DAPI (Roche, Basel, Switzerland; 10,236,276,001) for 5 min. The coverslips were washed extensively and mounted onto slides. Imaging of the cells was carried out using a LSM 780 Meta Confocal Microscope (Carl Zeiss, Oberkochen, Germany).

### Flow cytometry

Adult human RPE cells were trypsinized, incubated with human HLA-DR, CD14, CD34, and CD45 (R&D Systems, Minneapolis, MN, USA) for 30 min at 26 °C, washed, and then incubated with Cy5-conjugated goat anti-mouse IgM (Jackson Immunoresearch, West Grove, PA, USA) for 20 min at room temperature. Cells were analyzed on a FACS Aria 2 Cell Sorter (BD Biosciences, San Jose, CA, USA) with 10,000 events acquired for each sample. Data were analyzed with FlowJo software, a subsidiary of BD Biosciences.

### Tumor formation assay

ES and RPE cells were harvested, suspended in PBS, and injected subcutaneously into SCID mice (4–6 weeks old, Zhengzhou University, Permission No. *ShanhaiTech*, 20,170,012,003,062) (3 × 10^6^ cells per mouse). Four weeks (*n* = 6), 12 weeks (*n* = 7), and 24 weeks (*n* = 8) after the injection, animals were humanely sacrificed via isoflurane inhalation anesthesia prior to teratoma isolation. The tumors were then dissected and analyzed for the percentage of tumor formation in these mice. The paired samples *t* test was used to analyze the tumor formation data. All of the animal handling and procedures were approved by the Institutional Animal Care and Use Committee at Henan Provincial People’s Hospital, Zhengzhou, China.

### High performance liquid chromatography (HPLC) analysis

For HPLC sample preparation, 1 × 10^6^ RPE cells were homogenized in 200 μl of 0.4 M perchloric acid. Homogenates were centrifuged at 12,000 rpm for 20 min at 4 °C. HPLC analysis were performed using a HPLC system with electrochemical detection (Eicom HTEC-500, Kyoto, Japan) coupled with a Uniget C-18 reverse phase microbore column as the stationary phase (BASi, West Lafayette, IN, USA; cat no. 8949). The 1-l mobile phase consisted of 8.84 g citric acid monohydrate, 10 g sodium acetate anhydrate, 220 mg sodium octane sulfonate, 5 mg EDTA-2Na, and 200 ml methanol. The flow rate was 0.4 mg/ml, and 10 μl of the sample supernatant was injected directly into the HPLC for analysis. Dopamine standards, DOPAC (Sigma-Aldrich, St. Louis, MO, USA), were used to quantify and identify the peaks on the chromatographs.

### One-way mixed lymphocyte reaction (MLR)

A MLR assay was performed as described previously (Bromelow et al., 2001; Waldner et al., 2018). Briefly, peripheral blood mononuclear cells (PBMC) were prepared from heparinized venous blood taken from healthy adult volunteers. The blood was diluted 1:1 with RPMI-1640 medium (Life Technologies, Paisley, UK) and purified by Ficoll-Paque (GE). Peripheral blood lymphocytes (PBL) were purified from the PBMC preparation by the removal of plastic-adherent cells during culture at 37° for 1 h in a horizontal 35 cm^2^ flask (Corning, Stone, UK). Responder cells were isolated from the PBMCs according to the protocol mentioned above. PBMCs from other donors (PBL#) or human ES cells or MCF10A cells or RPE cells or induced dopamine-producing (iDA) cells irradiated with 3000 rads were used as stimulator cells. Responder cells (1 × 10^4^) and stimulator cells (1 × 10^4^) were co-cultured in 100 μl in 96-well plates at 37 °C in 5% CO_2_. After culturing for 96 h, a CCK8 assay was performed in order to evaluate the proliferation of the responder cells. Results were expressed as a stimulation index (SI). The SI was calculated using the following equation: SI = OD of responder cells in wells with stimulator cells added/OD of the same responders in wells containing responder cells only.

### PD monkey model

Two adult (2-year-old) male cynomolgus monkeys (*Macaca fascicularis*) were used for this study. The monkeys were cared for and handled according to the Guidelines for Animal Experiments of China. In order to create the PD model, the animals were injected intravenously with MPTP hydrochloride (0.4 mg/kg as a free base; Sigma-Aldrich, St. Louis, MO, USA) twice a week until we observed persistent PD symptoms, such as tremors, bradykinesia, and impaired balance. Stable symptoms of PD were observed before an animal was used for the experiments. The behavior of the animals (before modeling, after modeling and after transplantation) was evaluated according to a rating scale for monkey PD models. This scale rates nine items: alertness (0–3); head checking movement (0–3); eye blinking and movement (0–3); posture (0–3); balance (0–3); motility at rest (0–3); reactive motility to external stimuli (0–3); walking (0–3); and tremors (0–3). Normal and total maximum score is 27, and the minimum score is 0. Three observers blind to the treatment, have evaluated the behavior of the animals before modeling, after modeling and after cell transplantation. All of the animal handling and procedures were approved by the Institutional Animal Care and Use Committee at Henan Provincial People’s Hospital, Zhengzhou.

### Method of transplantation

At the time of the surgical procedure, PD model monkeys were placed in a stereotactic frame after receiving local anesthesia (with a continuous intravenous infusion of propofol (6 mg/kg/h), and their respiration state was adjusted to normal range PaO2 > 100 mmHg, PaCO2 ∼ 35 mmHg) by altering the ventilation rate, and the putamen was visualized with a high-field-strength MRI (1.5 T) using a fast spin-echo sequence. Axial images were obtained using contiguous 3-mm sections extending from below the putamen to above the caudate in a plane. Target sites for implantation were located in the posterior putamen. PD model monkeys were then transferred to the operating room. After local anesthesia, a burr hole was made in the calvarium at 5 mm anterior to the coronal suture and 25 mm from midline. A total of 1 × 10^6^ hRPE cells or HAE cells in 200 μL were deposited unilaterally in the posterior putamen. The behavior of the animals was evaluated 8 weeks after transplantation

### Statistics

The ANOVA statistical test was used for analysis of stimulation index, CCK8, cell cycle, cell adhesion, living cell ratio and clinical training scores. The Student’s *t* test was used for the comparison the teratoma data. For all tests, a *p*-value of < 0.05 was considered significant.

## Results

### Isolation and characterization of RPE cell lines from adult human eye tissues

**Fig. 1:**
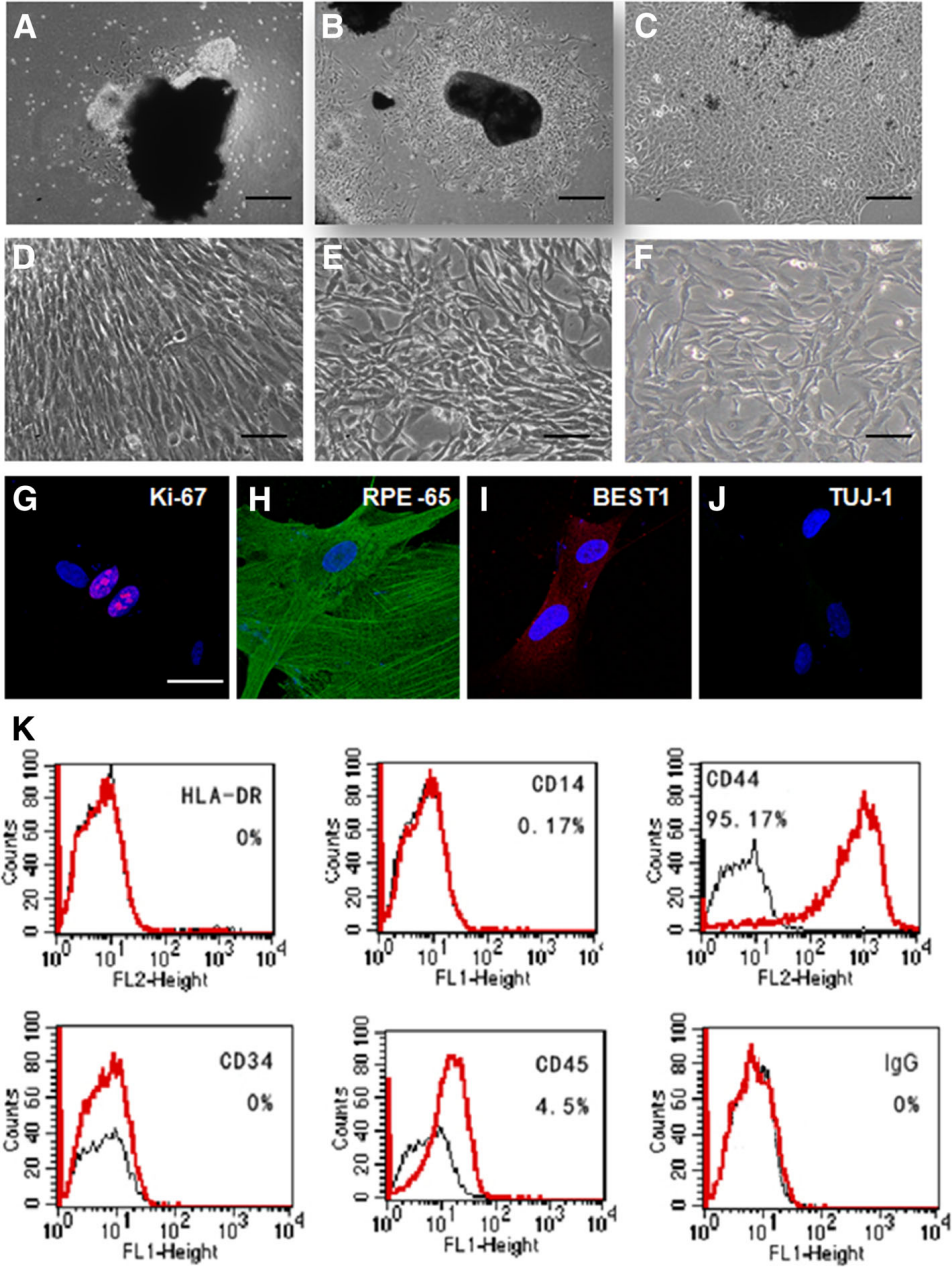
Isolation and characterization of cells from adult human RPE tissues. (**a**-**c**) Phase-contrast microscopy (A) of cultured RPE monolayer tissues. A small number of cells migrated out from the edge of the tissues. **b**, **c** While the cell layer expanded, the center tissues shrunk. The cell layer exhibited classical epithelial morphology with flat cell bodies possessing processes extending out from the cell body (scale bar: 100 μm). **d**-**f** Representative microscopy images of RPE cells at passages P1 (**d**), P10 (**e**), and P20 (**f**) (scale bar: 50 μm). **g**-**j** Characterization of cultured RPE cells by immunofluorescent staining using an antibody against Ki67 for proliferation (**g**), RPE65 (**h**) and BEST1 (**i**) for retinal pigmented epithelium, and Tuj1(**j**) for pan-neural cells (scale bar: 20 μm). **k** Flow cytometry analysis of the expression of HLA-DR, CD14 for endothelial, CD34 and CD45 for hematopoietic, and CD44 for proliferative progenitor cells

RPE tissue was isolated from frozen posterior eye tissue, and tissue debris was cultured in serum-free conditional media. Twenty-two hours after culturing, it was observed that cells migrated out of the tissue (Fig. 1a), and a single cell layer formed around the debris around 42–72 h (Fig. 1b and c). Both the morphology and proliferation rate became stable at 3 passages, and cells were depigmented at this stage. Cells were maintained for up to 20 passages (P20) (Fig. 1d,e and f) for future studies.

P20 RPE cells were used to investigate the expression of cell proliferation and cell fate markers. These cells were positive for Ki67, a marker for cell proliferation (Fig. 1g) and for RPE65 and BEST1, markers for retinal pigment epithelium (Fig. 1h and i). Expression of Tuj1, a pan-neuronal marker (Fig. 1j), was not detected at this stage. With regard to cell surface markers, flow cytometry analysis indicated that RPE cells were negative for HLA-DR, CD14, CD34, and CD45, which are surface markers for leukocyte, endothelial, and hematopoietic cells, respectively. In contrast, they were positive for CD44 (more than 95%), a marker for progenitor cells in variety of tissues (Fig. 1k).

### Evaluation of tumorigenicity and immunogenicity for cultured RPE cells

**Fig. 2:**
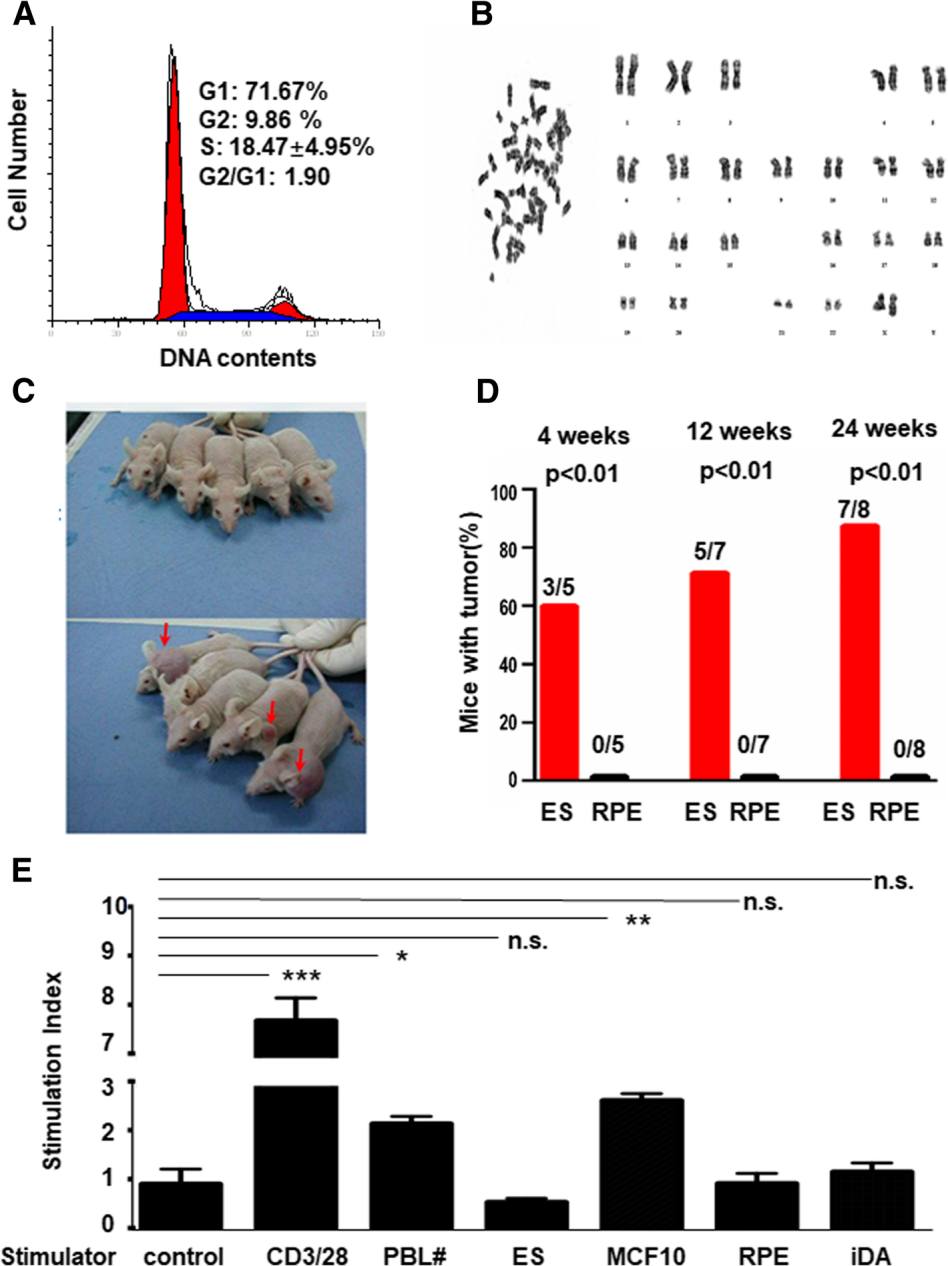
Evaluation of the safety and immunogenicity of cultured RPE cells. **a** Representative cell cycle progression of the P20 passage of RPE cells. **b** Representative G-banding analyses of karyotype for P20 passage RPE cells. **c** A picture of tumor formation in SCID-nude mice 4 weeks after injection of RPE (top panel) and ES cells (bottom panel) (*n* = 5 animals for each condition) Mice with tumors were arrowed (bottom panel). **d** Statistical analysis of the percentage of SCID-nude mice with tumors that formed from the ES and RPE cells measured at 4 (*n* = 5 animals for each condition), 12 (*n* = 7 animals for each condition), and 24 (*n* = 8 animals for each condition) weeks after injection. **e** Measurement of hPBL to co-cultured CD3/28, mismatched individual hPBL, ES, MCF10A, RPE, and iDA by one-way mixed lymphocyte reaction. The results were presented as a stimulation index. *p* values were determined using one-way ANOVA. Data are expressed as mean + SD; *p < 0.01, **p < 0.001, ***p < 0.0001 n.s., not significant; *n* = 3)

To fully characterize the RPE cells, cell cycle progression and chromosome stability were measured. There was a high proportion of cells in S-phase (18.47 ± 4.95%) (Fig. 2a). The cells exhibited normal human diploid karyotypes containing 22 pairs of autosomal chromosomes and one pair of X chromosomes (46, XX) (Fig. 2b). Tumorigenicity of RPE cells was further examined by teratoma formation. The same number of RPE cells or ES cells were subcutaneously injected into SCID-nude mice (Fig. 2c), and tumor-formation was measured at 4, 12 and, 12 weeks after injection. Tumor formation was observed in the mice grafted with ES cells at all three time points (Fig. 2d). However, no tumor formation was observed in the mice grafted with RPE cells at passage 20 (Fig. 2d).

To test the immunogenicity of RPE cells, a one-way mixed lymphocyte reaction (MLR) assay was used to measure ex vivo cellular immunity. As shown, both RPE cells and iDA cells failed to stimulate an immune response from co-cultured human peripheral blood lymphocytes (hPBL) cells (Fig. 2e). On the contrary, both the same number of PBL cells from mismatched individuals and MCF10A breast tumor cells stimulated a significant immune response (Fig. 2e).

### Chemically induced reprogramming of RPE cells into dopamine-producing cells

It is known that both SB431542, an inhibitor for transforming growth factor (TFG) β receptor, and CHIR99021, an inhibitor for glycogen synthase kinase 3 (GSK3), are required for inducing ES cells/iPS cells to midbrain neurons (Chambers et al., 2009; Doi et al., 2014; Kirkeby et al., 2012; Kriks et al., 2011; Perrier et al., 2004). Based on this knowledge, we looked for other chemicals (Additional file 1: Figure S1) that could improve the efficacy of the combination of SB431542 and CHIR99021 (Com SC) in the reprogramming of RPE cells into dopamine-producing cells. During normal development, midbrain dopaminergic neurons are generated from neurons in the floor plate. In this study, we defined an effective population as cells that expressed FOXA2 (a floor plate neuronal marker).

**Fig. 3:**
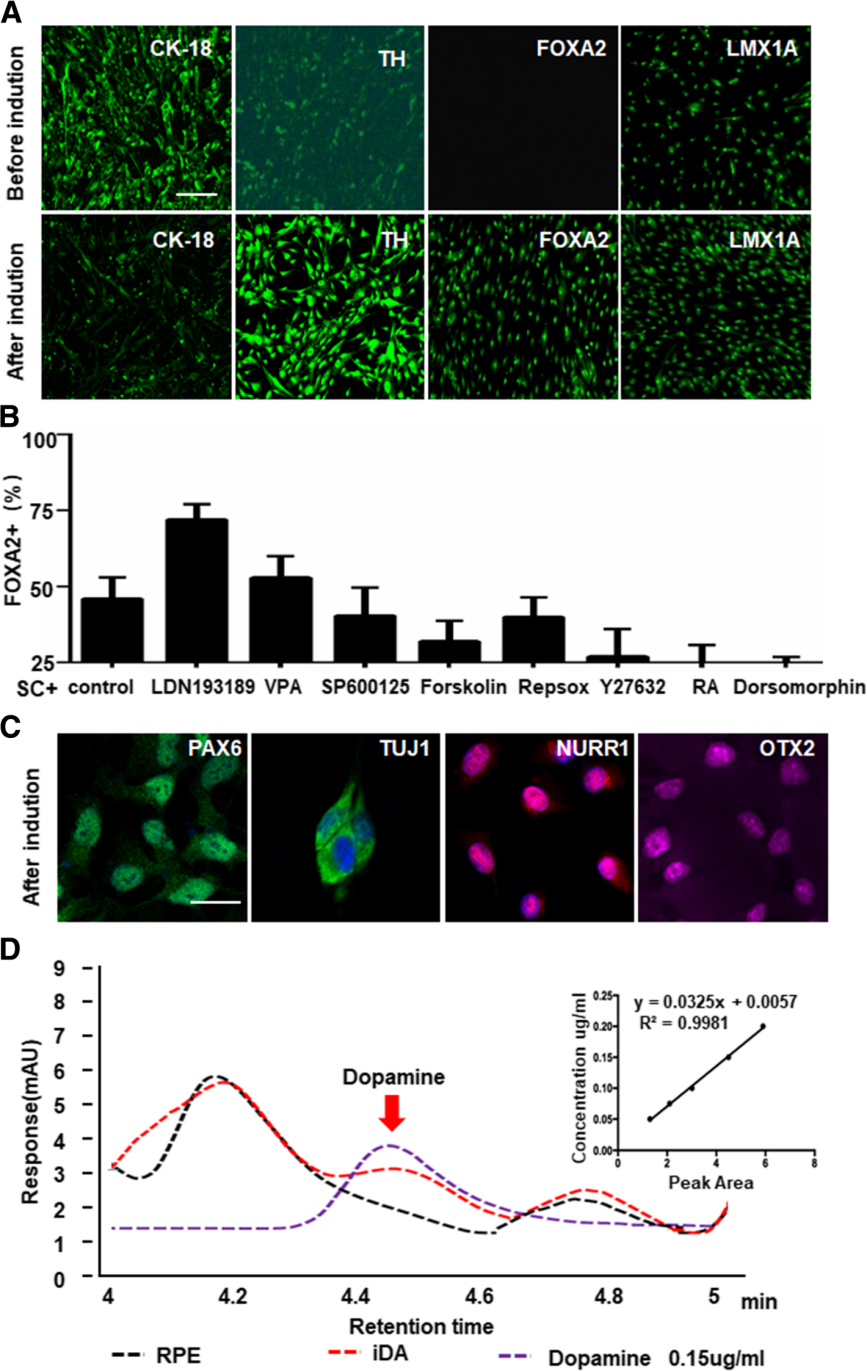
Chemically induced reprogramming of RPE cells into dopamine-producing cells. **a** Immunofluorescent staining of RPE cells before or 4 days after induction for CK-18, TH, FOXA2, and LMX1A, (scale bar: 100 μm). **b** Percentage of FOXA2^+^ cells in chemical induction systems with different small molecules. Com SC, combination of SB431542 and CHIR99021 was used as control. (Statistical analysis from Fig. S2A). Dates are expressed as mean + SD. **c** Chemically induced RPE cells expressed neuronal markers, including PAX6, NURR1, TUJ1, and OTX2 (scale bar: 20 μm). **d** Determination of dopamine by high-performance liquid chromatography (HPLC; red, the supernatant from cultured RPE cells after chemical induction; purple, 0.15 μg/ml dopamine standards; black, supernatant from cultured RPE cells before induction). Linear calibration curve of dopamine concentrations. The curve was plotted by integrated peak area ratio versus dopamine

As expected, seven days after incubation, Com SC induced RPE cells to express FOXA2 (Fig. 3a and Additional file 1: Figure S2A), an essential transcription factors for midbrain neurons. Moreover, Com SC, along with BMP4 signal inhibitor LDN193189, termed as Com LSC, more efficiently promoted the percentage of FOXA2 positive (Fig. 3b) and tyrosine hydroxylase (TH, the key enzyme for dopamine production) (Fig. 3a and Additional file 1: Figure S2B) positive cells than that of Com SC group. There were no significant differences in the percentage of these markers when induced by Com SC alone or along with other chemical small molecules (Fig. 3b). Com LSC group, epithelium-restricted marker, CK-18, was no longer detected (Fig. 3a) under the same induction conditions. In addition, immunofluorescence of PAX6, TUJ1, NURR1, and OTX2 all showed positive staining (Fig. 3c), suggesting that the induced cells became neuron-like cells. These neuron-like cells also expressed midbrain dopaminergic neuron markers, such as dopamine transporter (DAT) and VMAT2 (Additional file 1: Figure S3A). Additionally, some TH+ cells expressed markers for A9-type dopaminergic neurons of the nigrostriatal pathway, GIRK2 and Calbindin (Additional file 1: Figure S3B), suggesting that some reprogrammed dopaminergic-like cells exhibited a substantia nigra phenotype. More importantly, Com LSC- induced RPE cells were indeed able to produce dopamine at about 49.87 ± 17.5 ng per 10^6^ cells, as measured by HPLC (Fig. 3d).

### Human RPE cells differentiated into dopaminergic-like cells in the brain of monkeys after being transplanted into the caudatoputamen

**Fig. 4:**
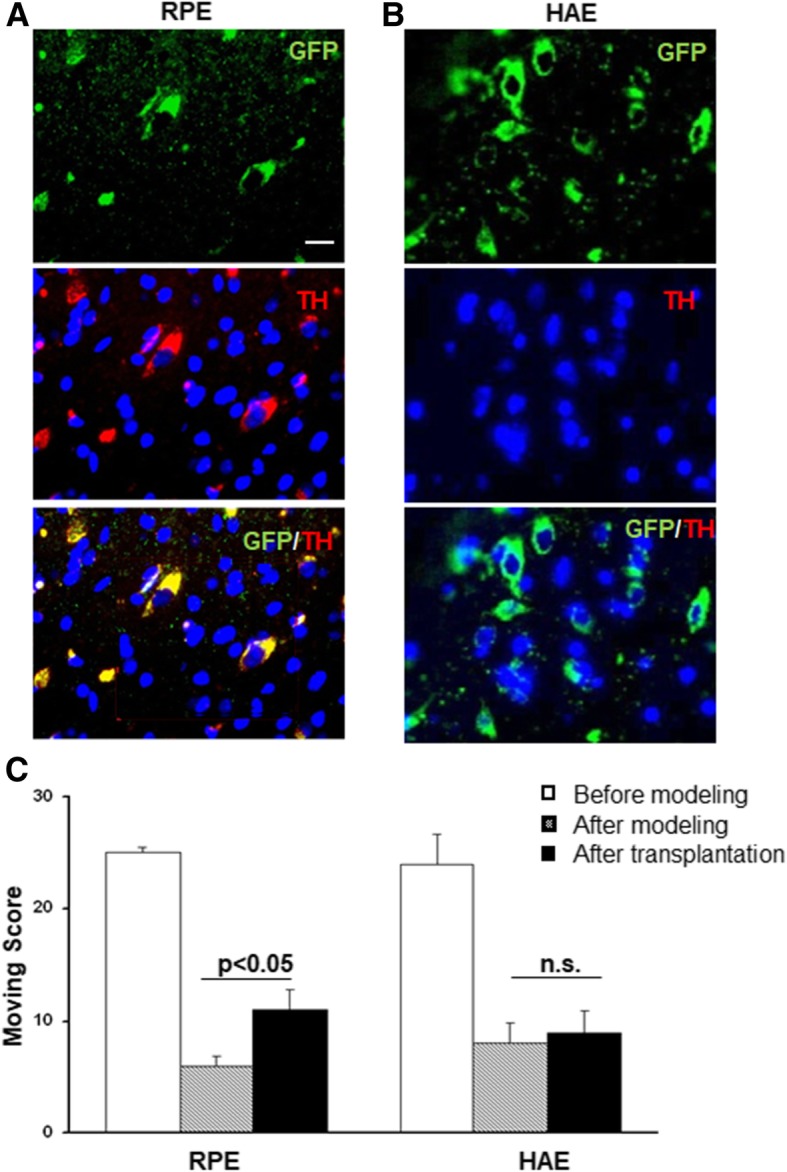
Human RPE cells differentiated into dopaminergic-like cells in the brains of monkeys after being transplanted into the striatum. **a**-**b** Immunofluorescent staining of tyrosine hydroxylase (red) in GFP-labeled human RPE cells (**a**) or HAE cells (**b**) (scale bar: 10 μm). **c** Spontaneous movements score for control, PD, or PD monkeys after transplantation with RPE cells (left panel) or HAE cells (right panel), data are expressed as mean + SD

The most valuable model of PD available is the primate model treated with MPTP, which is frequently used to study responses to new treatments (Imbert et al., 2000). To investigate whether RPE cells could differentiate into dopaminergic–like cells in the brain, GFP-labeled RPE or human amniotic epithelial (HAE) cells were injected into the caudate putamen of the brains of monkeys with PD, which exhibited severe motor behavior deficiency. After transplantation, both human RPE (Fig. 4a) and HAE cells (Fig. 4b) grafted well into the recipient brain (green in Fig. 4a, b). However, only grafted human RPE cells differentiated into TH+ cells (red and yellow in Fig. 4a). This is consistent with the observation that the clinical conditions were significantly improved in PD monkeys grafted with RPE cells (Fig. 4c), but not HAE cells (Fig. 4c).

## Discussion

PD affects about 1% of the population over 60 years of age. Cell therapy may provide a cure for this disease in the future, but it is crucial to determine what cells are reliable and what differential stages are suitable for transplantation. Successful cell therapy for PD should merit the following properties: 1) survival of grafted cells in host tissue; 2) differentiation of grafted cells into DA neurons to produce dopamine in a regulated manner; 3) amelioration of the behavioral deficits in patients with minimum side effects; 4) low immunogenicity response; and 5) low risk of tumorigenicity. However, there is currently no cell type that meets all of these standards. Although fetal tissues have been applied in animal experiments with satisfying results, they may not be able to enter clinical use due to ethical concerns. The current research overcame ethical challenges by successfully establishing RPE cell lines from adult eye tissues with informed consent. In addition, results of the current study provided evidence that RPE cells have minimal immunogenicity and little risk for tumorigenicity. These results suggest that RPE cells are a good alternative resource for cell therapy. The major challenge, however, is how to efficiently induce cells into dopamine-producing cells.

Based on the previous studies that reported that the combination of SB431542 and CHIR99021 were able to induce ES/iPS cells to become neuronal cells (Chambers et al., 2009; Doi et al., 2014; Kikuchi et al., 2017; Kirkeby et al., 2012; Kriks et al., 2011; Perrier et al., 2004), here, we have established a novel approach to efficiently induce RPE cells into dopamine-producing cells by the combination of SB431542, CHIR99021, and LDN193189 (Com LSC), an inhibitor of BMP4 signaling. This is supported by the fact that inhibition of BMP4 signaling facilitates neural induction in vitro (Chambers et al., 2009; Gerrard et al., 2005). In addition, Com LSC-induced RPE cells could produce dopamine as high as about 35.8 ng per 10^6^ cells. This concentration of dopamine reached a level similar to that reported for RPE cells treated with PPX and ROP conditional medium (Ming et al., 2009).

Most preclinical studies for cell therapy were performed in small animals, such as mice or rats, which cannot always translate therapeutic efficacy for human patients. In the present study, we transplanted RPE cells, in direct comparison with other cells, into the brain of monkeys with PD in order to address the therapeutic effect. We observed that only grafted RPE cells, but not other cells, improved the clinical conditions of monkeys with PD. Furthermore, we provided evidence that only grafted RPE cells became TH-positive cells, suggesting they had differentiated into dopaminergic neurons. This is the first report of a cell therapy study by stereotaxic transplantation of RPE cells into the caudate putamen of the brain of monkeys with PD.

## Conclusions

In conclusion, here we have successively established RPE cell lines from adult human eye tissues. Such cells can be chemically reprogrammed into dopamine-producing cells in vitro. After being grafted into the brain caudate putamen of chemically induced PD monkeys, RPE-grafted PD monkeys showed significant improvement of clinical conditions, due to the fact that some transplanted RPE cells became positive for tyrosine hydroxylase, suggesting the cells had differentiated into dopaminergic neurons. Despite all these observations, we are still unable to conclude that such RPE cells can be used for treating human PD patients due to the limited numbers of tested monkeys, as well as the limited length of observation time after transplantation in the study. A double-blind trial using Com LSC-induced RPE cells in large numbers of big animals, or PD patients in clinical trials, is necessary to address the therapeutic efficacy for RPE cells. Nonetheless, our study suggests that human adult RPE cells ban be an alternative cell source to treat PD in the future.

## Additional file

Additional file 1: **Figure S1.** Chemical compounds reported to induce neural cell differentiation and reprogramming. **Figure S2.** Chemically induced reprogramming of RPE cells into dopamine-producing cells. (A) Immunofluorescent staining of chemically induced RPE cells for FOXA2 (scale bar: 50 μm).(B) Immunofluorescent staining of chemically induced RPE cells for TH (scale bar: 50 μm). (Empty, without any chemical small molecules; S, SB431542; C, CHIR99021; Com SC, combination of SB431542 and CHIR99021; Com LSC, combination of LDN193189, SB431542 and CHIR99021.). **Figure S3.** Characteristics of chemically induced dopamine-producing cells. (A) Immunofluorescent staining of chemically induced RPE cells for VMAT2 or DAT (scale bar: 50 μm). (B) Immunofluorescent staining of chemically induced RPE cells for GRIK2 or Calbindin along with TH (scale bar: 50 μm). (DOCX 9774 kb)

## Abbreviations

BMP4: Bone morphogenetic protein 4
Com LSC: Combination of LDN193189, SB431542 and CHIR99021
Com SC: Combination of SB431542 and CHIR99021
DA: Dopamine
DBS: Deep brain stimulation
ES: Embryonic stem
GPi: Globus pallidus internus
GSK3: Glycogen synthase kinase 3
HAE: Human amniotic epithelial
hPBL: Human peripheral blood lymphocytes
HPLC: High-performance liquid chromatography
iDA: induced dopamine-producing cell
iPS: induced pluripotent stem cells
MCF: Michigan Cancer Foundation
MLR: Mixed lymphocyte reaction
MPTP: 1-methyl-4-phenyl-1,2,3,6-tetrahydropyridine
NSCs: Neural stem cells
P20: 20 Passages
PBL: Peripheral blood lymphocytes
PBMC: Peripheral blood mononuclear cells
PD: Parkinson’s disease
PPX: Pramipexole
ROP: Ropinirole
RPE: Retinal Pigment Epithelium
SCID: Severe combined immunodeficiency
SI: Stimulation Index
TFG: Transforming growth factor
TH: Tyrosine hydroxylase
