# Supplementary material for: Differentiation of adult human retinal pigment epithelial cells into dopaminergic-like cells in vitro and in the recipient monkey brain
Source: Mol Med. 2019 Mar 29;25:9. doi: 10.1186/s10020-019-0076-3 (PMC6440004; doi:10.1186/s10020-019-0076-3)
Supplement: Supplementary file 1 — Figure S1. Chemical compounds reported to induce neural cell differentiation and reprogramming. Figure S2. Chemically induced reprogramming of RPE cells into dopamine-producing cells. (A) Immunofluorescent staining of chemically induced RPE cells for FOXA2 (scale bar: 50 μm).(B) Immunofluorescent staining of chemically induced RPE cells for TH (scale bar: 50 μm). (Empty, without any chemical small molecules; S, SB431542; C, CHIR99021; Com SC, combination of SB431542 and CHIR99021; Com LSC, combination of LDN193189, SB431542 and CHIR99021.). Figure S3. Characteristics of chemically induced dopamine-producing cells. (A) Immunofluorescent staining of chemically induced RPE cells for VMAT2 or DAT (scale bar: 50 μm). (B) Immunofluorescent staining of chemically induced RPE cells for GRIK2 or Calbindin along with TH (scale bar: 50 μm). (DOCX 9774 kb) [file 10020_2019_76_MOESM1_ESM.docx]

**Supplemental material**

Supplemental Figure 1.
Chemical compounds reported to induce neural cell differentiation and reprogramming.

Supplemental Figure 2. Chemically induced reprogramming of RPE cells into
dopamine-producing cells.
(A) Immunofluorescent staining of chemically induced RPE cells for FOXA2 (scale bar: 50 μm).(B) Immunofluorescent staining of chemically induced RPE cells for TH (scale bar: 50 μm).

(Empty, without any chemical small molecules; S，SB431542；C, CHIR99021; Com SC, combination of SB431542 and CHIR99021; Com LSC, combination of LDN193189, SB431542 and CHIR99021.).

Supplemental Figure 3. Characteristics of chemically induced dopamine-producing cells.
(A) Immunofluorescent staining of chemically induced RPE cells for VMAT2 or DAT (scale bar: 50 μm). (B) Immunofluorescent staining of chemically induced RPE cells for GRIK2 or Calbindin along with TH (scale bar: 50 μm).

**Response to reviewer’s comments**

**Reviewer #2:**

**Comment:** It would be helpful for the reader if in Figure 2C (bottom panel) arrows would point to the mice with tumors.

**Response:** We have done so in the revised Figure 2C.

**Comment:** How were the tumors analyzed? This is not sufficiently addressed in the revision, e.g. was histology performed, was there any difference in tumor size between animals or was immune suppressants used? Please provide more information.

**Response:**

To address the tumorigenicity of RPE cells, we have compared the tumor formation in the mice injected with RPE cells or ES cells. Same number of cells (3×10^6 cells per mouse) were subcutaneously injected into SCID-nude mice (ShanhaiTech, 20170012003062) without any immune suppressants (We have added this information in revised Manuscript)

Tumor formation was observed only in the mice grafted with ES cells (Fig. 2c, d) but not in the mice grafted with RPE cells (Fig. 2c, d). Since we do not predict that RPE cells could form tumors, in this study, we cared more about how may mice got tumors after grafted with either ES cells or RPE cell. Calculated 24 weeks after grafted cells, for example, seven in eight mice grafted with ES cells got tumors, whereas no mice got tumor that had been grafted with RPE cells. The Student's *t*-test was used for analysis.

Since no tumor formed in mice grafted with RPE cells, we only analyzed histological characters of tumors from the mice grafted with ESCs (Fig. 1). (Scale bar, 50 μm)

**Fig.1:**
Following the reviewer’s suggestion, we have revised the Figure 2C and figure legends as shown here:

Figure2：（C）A picture of tumor formation in SCID-nude mice 4 weeks after injection of RPE (top panel) and ES cells (bottom panel) (n = 5 animals for each condition) Mice with tumors were arrowed (bottom panel). (D) Statistical analysis of the percentage of SCID-nude mice with tumors that formed from the ES and RPE cells measured at 4 (n = 5 animals for each condition), 12 (n = 7 animals for each condition), and 24 (n = 8 animals for each condition) weeks after injection.

**Comment：**Statistical analyzes using Student's t-test are not appropriate for experiments shown in Figure 2E. The authors should rather use ANOVA.

**Response：**

We take this suggestion, and used ANOVA to analyze the stimulation index in Figure 2E.

The detailed statistical analyzes results are as below here.

According to the new statistical analyzes we have provided the new Figure 2E in revised manuscript as shown in the following:

Figure 2E: Measurement of hPBL to co-cultured CD3/28, mismatched individual hPBL, ES, MCF10, RPE, and iDA by one-way mixed lymphocyte reaction. The results were presented as a stimulation index. p values were determined using one-way ANOVA. Data are expressed as mean + SD; *p<0.01, **p<0.001, ***p<0.0001, n.s., not significant; n =3.

**Comment:** Figure legend of supplementary Figure 2 is still insufficient. Please explain in more detail what do you mean for example with control, S, C and SC? Moreover, the term Com LSC is unclear. You wrote in result section '…Com SC, along with BMP4 signal inhibitor LDN193, termed as Com LSC, more efficiently promoted the percentage of FOXA2 positive (Fig. 3b) and tyrosine hydroxylase (TH, the key enzyme for dopamine production) (Fig. 3a and Fig S2B) positive cells.' but no information of Com LSC is provided in abbreviations and in legend and corresponding Figures. Finally, as compared to which group Com LSC promoted more efficiently FOXA2 expression? (see comment above). Please clarify. For the new Figure 3B authors mention that new statistical analyzes were added, however, no such statement can be found. Moreover, are the values expressed as mean + SD or sem?

**Response:** It was our fault of presenting the confused interpretation in the previous manuscript. During the normal development, midbrain dopaminergic neurons are generated from neurons in the floor plate. In this study, we defined an effective population as cells that expressed FOXA2. To evaluate the efficiency of chemical compounds in inducing neural cells differentiation and reprogramming, we have used the Com SC group as control and analyzed the cell ratio of FOXA2+ in all inducing system. As shown, combination SB431542 and CHIR99021 and LDN193189, termed as Com LSC, were selected based on the induction ratio of FOXA2+. We have relabeled the figures in supplementary Figure 2 and revised them in our manuscript. In addition, we also added new statistical analyzes in Figure 3B.

**Supplementary Figure 2**

Supplemental Figure 2. Chemically induced reprogramming of RPE cells into

dopamine-producing cells.

(A) Immunofluorescent staining of chemically induced RPE cells for FOXA2 (scale bar: 50 μm). (B) Immunofluorescent staining of chemically induced RPE cells for TH (scale bar: 50 μm).

(Empty, without any chemical small molecules; S，SB431542；C, CHIR99021; Com SC, combination of SB431542 and CHIR99021; Com LSC, combination of LDN193189, SB431542 and CHIR99021.).

**Figure 3B**

(B) Percentage of FOXA2+ cells in chemical induction systems with different small molecules. Com SC, combination of SB431542 and CHIR99021 was used as control. Dates are expressed as mean + SD (Statistical analysis from Fig S2A).

**Manuscript:**

“As expected, seven days after incubation, Com SC induced RPE cells to express FOXA2 (Fig. 3a and Fig S2A), an essential transcription factors for midbrain neurons. Moreover, Com SC, along with BMP4 signal inhibitor LDN193189, termed as Com LSC, more efficiently promoted the percentage of FOXA2 positive (Fig. 3b) and tyrosine hydroxylase (TH, the key enzyme for dopamine production) (Fig. 3a and Fig S2B) positive cells than that of Com SC group. There were no significant differences in the percentage of these markers when induced by Com SC alone or along with other chemical small molecules (Fig. 3b).”

**Abbreviations**

Com LSC: combination of LDN193189, SB431542 and CHIR99021.

**Comment：** The part of the PD in vivo models needs further information. So it is unclear how many monkeys were included in the study. At which time point after the transplantation were the animals behaviorally investigated? What means the sentence '…representative spontaneous movements score…' and how are the data presented. Please clarify.

**Response:**

**1**. Two (2-year-old) male cynomolgus monkeys (Macaca fascicularis) were used to create the PD model. After PD modeling, one of them was transplanted with human RPE cells and another was with HAE cells in the posterior putamen. **We have added this information in“PD Monkey Model" section in the revised manuscript.**

**2**. The behavior of the animals was evaluated 8 weeks after transplantation. **We have added this information in “Method of Transplantation " section in the revised manuscript.**

**3.** The behavior of the animals (before modeling, after modeling and after transplantation) was evaluated according to a rating scale for monkey PD models. This scale rates nine items: alertness (0 – 3); head checking movement (0 – 3); eye blinking and movement (0 – 3); posture (0 – 3); balance (0 – 3); motility at rest (0 – 3); reactive motility to external stimuli (0 – 3); walking (0 – 3); and tremors (0 – 3). Normal and total maximum score is 27, and the minimum score is 0. Three observers blind to the treatment, have evaluated the behavior of the animals before modeling, after modeling and after cell transplantation. (mean + SD is represented. Statistical analysis was done by using analysis of variance (ANOVA).

**Comment：**Moreover, in Figure 4A histological photomicrographs are shown, however, no information is given how that was achieved, i.e. animal and tissue processing. Please also provide information on anesthesia.

**Response:** For histological studies, animals were perfused with 100 mM PBS and then 4% paraformaldehyde in PBS **following an intravenous infusion of Potassium Chloride (KCl) (75-150 mg/kg) after general anesthesia**. Brains were post-fixed for 1 day with 4% paraformaldehyde and then transferred to 10, 20 and 30% sucrose solution at 4 °C. The brains were sectioned at 40 μm thickness using a microtome. Histochemical analyses were carried out after permeabilization and blocking with phosphate buffered saline containing 5% bovine serum albumin for 30 minutes.

**General anesthesia:** with a continuous intravenous infusion of propofol (6 mg/kg/h).

**Euthanasia:** intravenous infusion of Potassium Chloride (KCl) (75-150 mg/kg) after general anesthesia.

**We have added this information in “Immunofluorescence Microscopy" section in revised manuscript.**

**Comment：**For the monkey brains it is needed to show co-localization for TH and DAT.

**Response ：**Regarding this issue, in fact, we had tried co-staining of TH with DAT many times in monkey brain tissues. However, we have failed to get good staining due to non-specific background signals in the monkey tissues.

We therefore performed co-staining of TH and DAT (Scale bars, 50*um*) in human cells, which had been *in vitro* chemically induced from RPE cells into midbrain dopaminergic-like cells (see Figure above).

**Minor points:**

**1.** Scale bar is missing in suppl. Figure 3B.

We have added the scale bar in revised manuscript.

**2.** Please indicate from which animal species the antibodies were generated, e.g. mouse or rabbit anti-pax6?

Thank you for your advice. We have provided in revised manuscript.

**3.** In Figure 2 data are presented as mean + SD and not as mean +/- SD.

**Changed.**
